# Review of an Influenza Surveillance System, Beijing, People’s Republic of China

**DOI:** 10.3201/eid1510.081040

**Published:** 2009-10

**Authors:** Peng Yang, Wei Duan, Min Lv, Weixian Shi, Xiaoming Peng, Xiaomei Wang, Yanning Lu, Huijie Liang, Holly Seale, Xinghuo Pang, Quanyi Wang

**Affiliations:** Beijing Center for Disease Prevention and Control, Beijing, People’s Republic of China (P. Yang, W. Duan, M. Lv, W. Shi, X. Peng, X. Wang, Y. Lu, H. Liang, X. Pang, Q. Wang); University of New South Wales, Sydney, New South Wales, Australia (H. Seale)

**Keywords:** Influenza, viruses, surveillance, early warning, China, research

## Abstract

In 2007, a surveillance system for influenza-like illness (ILI) and virologic data was established in Beijing, China. The system tracked ILI and laboratory-confirmed influenza in 153 general hospitals from September 1, 2007, through April 30, 2008. To analyze the ILI surveillance data (weekly ILI rates and counts) and the effectiveness of the system, we used the US Centers for Disease Control and Prevention Early Aberration Reporting System. The data indicated that the highest rate of influenza isolation and the highest ILI count occurred in the first week of 2008. The system enabled us to detect the onset and peak of an epidemic.

## Abstract 2

This system enabled detection of the onset and peak of an epidemic.

Surveillance systems in Beijing, People’s Republic of China, play a pivotal role in the detection of seasonal influenza. They enable the onset and the peak of an influenza epidemic to be reported in a timely and accurate manner. These systems may be critical to monitoring future emerging aberrant situations, such as an influenza pandemic.

Since 1997, >400 human cases of infection with avian influenza virus A (H5N1) have been documented worldwide, with death rates of ≈60% (*1*). Of concern is that these influenza A viruses might undergo the genetic changes of antigenic drift into novel pathogenic forms (*2*), triggering human influenza pandemics (*3*). Recently, the World Health Organization (WHO) raised the influenza pandemic alert to level 6 because of the emergence of the influenza A pandemic (H1N1) 2009 virus (*4*). Experts at WHO believe that “the world is now closer to another influenza pandemic than at any time since 1968” (*5*).

An influenza surveillance program, consisting of disease and virologic data collection, aims to assist in the early detection of influenza, help define the distribution of influenza in the community, and provide timely information about circulating strains. These data, in turn, can be used to analyze geographic, temporal, and biologic differences in circulating influenza strains and assist in monitoring for emerging unusual or critical situations, such as a pandemic (*6*–*8*). This information can guide the crucial process of strain selection for vaccine development and other prevention and control strategies (*7*), as well as aid influenza diagnosis and enhance patient care (*9*–*12*).

To use data from a surveillance system efficiently, however, public health professionals need suitable and robust aberration detection methods. The Early Aberration Reporting System (EARS) pioneered by the US Centers for Disease Control and Prevention (CDC; Atlanta, GA, USA) was initially a method for monitoring bioterrorism events, but it has evolved into a tool that also can be used to monitor naturally occurring outbreaks and seasonal diseases. Nonhistorical methods based on a positive 1-sided cumulative sum (CUSUM) calculation in EARS can analyze the data without long-term background data (*13*,*14*).

In 2007, a surveillance system for influenza-like illness (ILI) and virologic data was established in Beijing. This system tracks ILI and laboratory-confirmed influenza in 153 general hospitals throughout Beijing. We describe the surveillance system, the surveillance data accumulated during the 2007–08 influenza season, and the performance of the early warning system.

## Methods

Beijing is located in the temperate zone of the Northern Hemisphere, where influenza typically peaks seasonally once each year (*15*). Hospitals in Beijing are classified into 3 levels, depending on their size and the techniques, equipment, and staff available (Table 1) (*16*). In Beijing, patients with ILI traditionally seek medical attention at their local hospitals rather than at private clinics.

**Table 1:** Three levels of hospitals in Beijing, People’s Republic of China

Level	Description
1	Services include medical treatment, prevention, healthcare, and rehabilitation for a community with a population <100,000 persons
2	Services include medical treatment, prevention, healthcare, and rehabilitation for multiple communities (population >100,000 persons
3	Regional healthcare facility with specialized high-level healthcare services for several districts

### Surveillance for ILI and Virologic Data

Influenza surveillance was performed from September 1, 2007, through April 30, 2008. In this system, ILI surveillance was conducted in the outpatient and emergency clinics of internal medicine and pediatric wards of 153 hospitals: 29 were level 1, 71 were level 2, and 53 were level 3.

Under the system, participating referral doctors were required to diagnose ILI by using a strict ILI definition (fever >38°C, either cough or sore throat, and no other laboratory-confirmed evidence) (*7*) and to record the number of ILI consultations by age group (i.e., 0–4 years, 5–14 years, 15–24 years, 25–59 years, and >60 years) on a fixed form daily. These data were entered daily into the Beijing Monitoring and Early Warning System for Infectious Diseases in Hospitals by designated hospital staff.

Fourteen hospitals from 6 districts were selected as sites for collecting specimens. Pharyngeal swab specimens from the ILI case-patients (within 3 days of symptom onset from patients who had not received antiviral drugs) were collected from the hospitals by district CDC staff. The specimens were transported to the correspondent laboratories in viral transport medium at 4°C for subsequent isolation and identification. Six independent laboratories in different districts participated in the collaborative laboratory network.

Weekly laboratory surveillance data were used as the approved standard estimate to measure the onset of an influenza epidemic. By monitoring the rate of positive isolations, changes in the activity of influenza virus were tracked. The positive isolation rate and the maximum weekly positive isolation rate were compared to determine the week the epidemic began. Because these rates change yearly, a fixed rate could not be regarded as the threshold. We used the following standard to ascertain the onset week: if the positive isolation rate in any given week exceeded 40% of the maximum weekly positive isolation rate in the overall influenza season, this week was then considered the onset week of the influenza epidemic (*17*).

### EARS and Early Aberration Detection of ILI Surveillance Data in Beijing

We used EARS-X v2.8 (*18*) to analyze the ILI surveillance data. The methods used in EARS are described elsewhere (*13*,*14*,*18*). Both the weekly ILI rates and ILI counts were analyzed by EARS-X. The nonhistorical method used in EARS consists of 3 algorithms, called C1-mild (C1), C2-medium (C2), and C3-ultra (C3). The terms mild, medium, and ultra refer to the level of sensitivity of the 3 alternative statistical methods, with C1 being the least sensitive and C3 the most sensitive. The thresholds for C1, C2, and C3 are based on a 1-sided positive CUSUM calculation (*13*,*14*,*18*). For C1 and C2, a warning is generated when the current count is greater than the baseline mean plus 3 SD. C1 uses data from 1 to 7 days before the current day for calculating the mean and SD, whereas C2 and C3 use data from 3 to 9 days in the past for calculating the mean and SD. For C3, the algorithm is based on a CUSUM calculation with an average run length time of 3 days. If the calculated value is >2, a C3 warning is produced. C1, C2, and C3 methods were simultaneously adopted for comprehensive analysis.

To determine the onset of the influenza epidemic, we established a standard based on warings in EARS (C1, C2, and C3). According to the difference of the sensitivities of C1, C2, and C3, the standard incorporated the following situations: 1) when a C1 warning was produced in a given week and the value of the following week was greater than that of the selected week, the selected week was the week of onset; or 2) when a C2 warning was produced in a given week and a C1 or C2 warning was generated in the following week, the following week was the week of onset; or 3) when a C3 warning was produced in a given week, and the following consecutive 2 warnings of C1, C2, or C3 were generated in the following 2 weeks, and the above 2 situations did not occur, the third week of the warning occurring was the week of onset.

## Results

### ILI Surveillance

**Figure 1:**
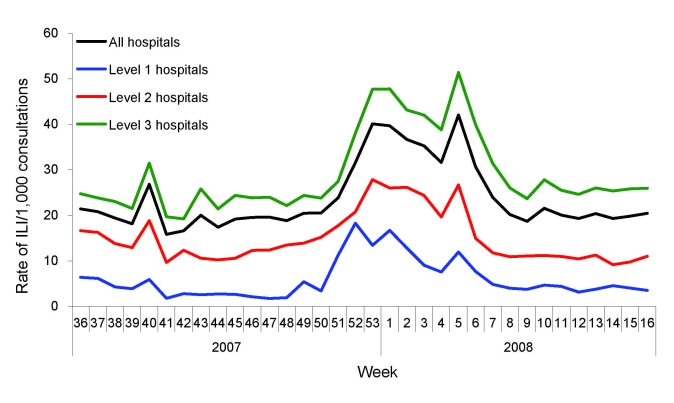
Weekly influenza-like illness (ILI) rates during the 2007–08 influenza season, Beijing, People’s Republic of China.

During the 2007–08 influenza season, the peak in ILI was identified on the basis of the weekly ILI surveillance data. For the 153 hospitals in the surveillance system, the highest weekly ILI rate during this season was 40.1 cases per 1,000 consultations in the week 53 of 2007 (Figure 1), 1 week earlier than when the ILI count peaked (18,203 cases in the first week of 2008, Figure 2). The highest weekly ILI rate and count are 1.7× and 2.0× as high as the average ILI rate (23.8/1,000) and count (9,154 cases), respectively. After stratification by hospital levels, the trends for level 1, 2, and 3 hospitals were similar. The highest ILI count for all age groups except the >60 years group occurred in the first week of 2008. For the >60 years group the ILI count peaked in the third week of 2008 (Figure 3).

**Figure 2:**
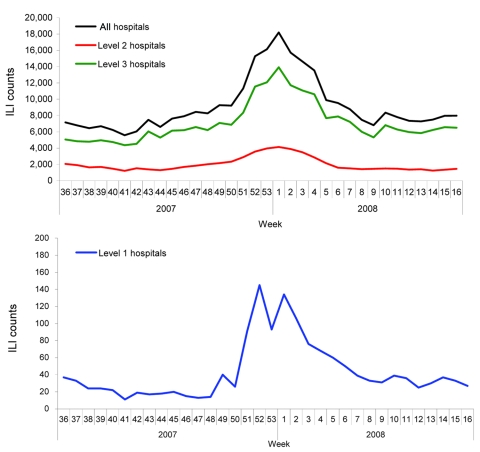
Weekly influenza-like illness (ILI) counts during the 2007–08 influenza season, Beijing, People’s Republic of China.

**Figure 3:**
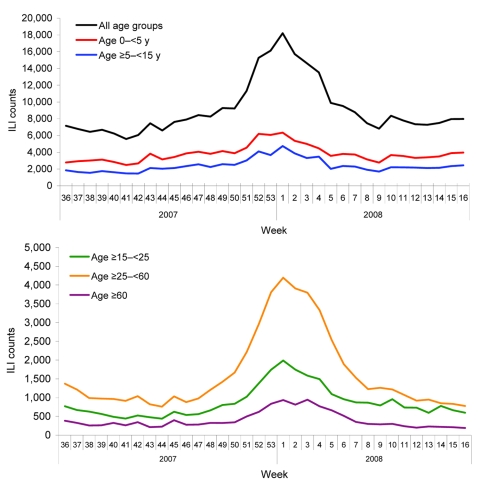
Weekly influenza-like illness (ILI) counts by age group during the 2007–08 influenza season, Beijing, People’s Republic of China.

### Virologic Surveillance

**Figure 4:**
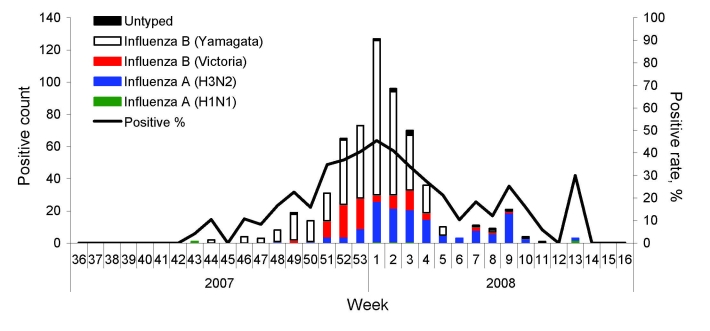
Weekly distribution of influenza isolates during the 2007–08 influenza season, Beijing, People’s Republic of China.

Pharyngeal swab samples were collected from 2,057 ILI case-patients during the influenza surveillance period. Thirty percent (n = 611) of these patients tested positive for influenza (type A, 151; type B, 450; untyped, 10). Overall, influenza B (Yamataga-lineage) was the dominant strain detected; however, late in the season, influenza A was isolated more frequently (Figure 4). On the basis of the 40% maximum weekly isolation rate (the gold standard indicating the onset of the influenza epidemic), week 49 of 2007 was considered the onset week (Figure 4).

### Early Warning by EARS of the Onset of an Influenza Epidemic

**Figure 5:**
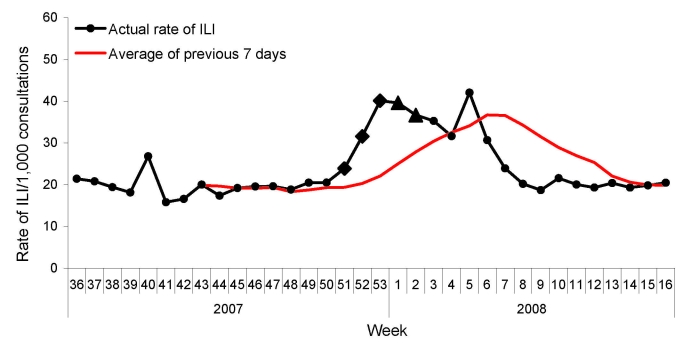
Weekly influenza-like illness (ILI) rates by Early Aberration Reporting System to detect the onset of the influenza epidemic during the 2007–08 season, Beijing, People’s Republic of China. Squares and triangles represent different alert situations—C1-mild (C1), C2-medium (C2), and C3-ultra (C3)—automatically generated by the reporting system: diamond, C1C3; triangle, C2C3.

**Figure 6:**
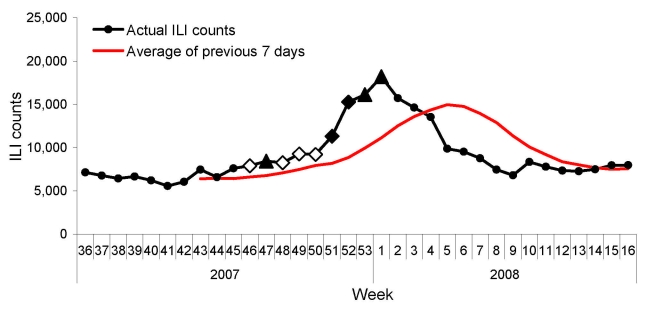
Weekly illness-like illness (ILI) counts by Early Aberration Response System to detect the onset of the influenza epidemic during the 2007–08 season, Beijing, People’s Republic of China. Triangles and diamonds represent different alert situations—C1-mild (C1), C2-medium (C2), and C3-ultra (C3)—automatically generated by the reporting system: triangle, C2C3; open diamond, C3; and solid diamond, C1C2C3.

According to the ILI rate data (Figure 5), a warning was first produced in week 51 of 2007, with C1, C2, and C3 warnings generated simultaneously. During week 52, the rate of ILI was greater than in the previous week; therefore, week 51 was set as the onset of the influenza epidemic, according to the standard described in the methods. From the ILI count data (Figure 6), 3 consecutive C3 warnings were produced between week 46 and week 48. According to these data, the influenza epidemic could have begun during week 48.

After stratifying the data by hospital level and age group, we determined the onset week from the ILI rate data for levels 1, 2, and 3 hospitals was week 51 of 2007 (Table 2). On the basis of the ILI count data, the onset week for level 1 hospitals was week 51, whereas for the level 2 and 3 hospitals, it was week 49. According to the ILI count data, onset weeks varied for different age groups.

**Table 2:** Onset of influenza epidemic as determined by the various ILI data during the 2007–08 influenza season, Beijing, People’s Republic of China*

Data	ILI rate			ILI count	
	Warnings meeting the standard	Onset week		Warnings meeting the standard	Onset week
Hospital level					
1	C1 in a given week and the greater following value	51		C1 in a given week and the greater following value	51
2	Consecutive 2 C2	51		Consecutive 2 C2	49†
3	C1 in a given week and the greater following value	51		Consecutive 3 C3	49†
Age group, y					
0–4	NA	NA		Consecutive 3 C3	48
5–14	NA	NA		Consecutive 2 C2	46
15–24	NA	NA		C1 in a given week and the greater following value	49†
25–59	NA	NA		C1 in a given week and the greater following value	49†
>60	NA	NA		C1 in a given week and the greater following value	51

*ILI, influenza-like illness; C1, C1-mild algorithm; C2, C2-medium algorithm; C3, C3-ultra algorithm; NA, not available. †Onset week, determined by the accepted standard based on 40% of the maximum weekly isolation rate, is the threshold

## Discussion

The 2007–08 influenza season in Beijing was mild, without a large documented epidemic or an outbreak. Influenza B predominated during the season in Beijing, which differed from the situations recorded in other countries and regions, including Europe and North America, where influenza A predominated (*19*). Influenza activity in Beijing may have been mild because of the epidemic characteristics of influenza B (*20*). From the data, we found that the highest rate of influenza isolation occurred in the first week of 2008, and the highest ILI rate (except the false high ILI rate in the fifth week of 2008, the Chinese Spring Festival week) occurred 1 week earlier (week 53 of 2007). However, on closer examination, we found that the week of the peak influenza isolation rate was actually almost identical to that of the highest ILI rate. Occurrence of the highest ILI rate in week 53 of 2007 resulted from the New Year’s holidays, which could have caused a decline in consultations during this period.

To understand the differences between the efficiencies of ILI rates and ILI counts in detecting the onset of an influenza epidemic, we analyzed both by using EARS. The onset week determined by the ILI rate (week 51) was 2 weeks later than the onset week (week 49) determined by a standard. However, in comparison, the onset week as determined by the ILI count was week 48, which still was close to the onset week determined by the reference standard but 3 weeks earlier than that determined by the ILI rate. When the data were stratified by hospital level and by age group, the onset weeks (week 46–week 49) as determined by the ILI count data were earlier than the week determined by the ILI rates (week 51), except for level 1 hospitals and the age group >60 years of age. One possible reason for the earlier onset detected by the ILI counts than by the ILI rates was the dependence of ILI rates on ILI counts and total consultations to the hospitals. Because humans are at high risk for many diseases during winter, consultations about other diseases also will increase. In this situation, it is optimal to consider the results for both the ILI rates and ILI counts simultaneously before any public health decisions are made.

Assessment of the timeliness and accuracy of determining the onset by hospital level showed that ILI count data from level 2 and 3 hospitals were more timely and accurate than were data from level 1 hospitals (week 49 rather than week 51). This result may be due to the tendency for level 2 and 3 hospitals to be larger and able to afford more comprehensive and reliable data than level 1 hospitals. Fluctuations in consultations could affect the accuracy of determining the onset of the influenza epidemic.

The inclusion of level 1 hospitals in the surveillance system may play a substantial role in the surveillance for human avian influenza and future pandemic activity. These hospitals were selected from smaller towns that have more poultry workers. These hospitals are likely to be the first destination for poultry workers who have ILI symptoms. If an atypical increase in the number of ILI consultations in these hospitals occurs in a short period, this increase could signal a need for further investigations and the institution of possible control measures. Poultry workers are a high-risk population for avian influenza (*21*,*22*), and ILI symptoms are the precursory symptoms of avian influenza (*23*). Earlier detection of ILI cluster cases in poultry workers may be helpful for finding clustered cases of avian influenza, human-to-human transmission cases, and the early stages of a pandemic. Therefore, ILI surveillance in these level 1 hospitals still should be continuously conducted.

When we stratified ILI count data by age group, we found the accuracy and timeliness of determining the onset of the influenza epidemic by the ILI count data were most efficient when using data in the age groups 15–24 years and 25–59 years. Although the onset weeks determined by the ILI data were earlier than week 49 for those 0–4-years and 5–14 years, this finding may be more likely to have resulted from respiratory syncytial virus circulation and infection. This virus can increase the number of emergency department visits and hospitalization in young children, and its season usually occurs before the annual influenza season (*24*,*25*). Another reason might be that the infection or reinfection rate in children by predominating influenza B virus was higher than that in adults (*26*), and thus more children were brought to doctors for treatment than adults. ILI data from the those >60 years of age were the last to give a warning for the epidemic onset (week 51) from the 5 age groups, perhaps because the ILI definition used may have been too strict to screen the influenza cases in the elderly. In elderly persons, fever and cough are relatively less common than they are in younger persons (*27*). In this system, monitoring ILI data in the young age groups is more efficient than monitoring influenza activity in the older age groups.

The ILI data obtained were the only means used to evaluate the efficacy of the surveillance system in detecting the onset of the influenza epidemic. We did not use the site-specific or age group–specific virologic data to ascertain whether true differences existed in the timing of influenza virus circulation between these specific groups. This omission is a limitation of this study.

In many developing countries, such as the People’s Republic of China, surveillance systems have only recently been implemented; therefore, classic statistical methods that require detailed historical data are not suitable (*28*–*31*). In these countries, programs such as EARS provide a suitable tool for detecting the aberration of data in surveillance systems without the need for historical data. It provides the most optimal and accessible tool, not only for seasonal surveillance but also for outbreak surveillance in developing countries. Furthermore, EARS is simple to grasp and practice and is freely downloadable from the US CDC website (http://emergency.cdc.gov/surveillance/ears)

The influenza surveillance system introduced in Beijing provided timely and accurate surveillance information that was consistent with data obtained from virologic surveillance for influenza. The system enabled us to detect the onset and peak of the epidemic. The ILI data from the larger hospitals may have afforded more valuable information for monitoring the onset of the epidemic than the data from smaller hospitals. However, given the current climate with avian influenza, it is crucial that the small provincial hospitals remain. In this situation, EARS was useful in the analysis of disease surveillance data, giving us the opportunity to undertake the surveillance without any historical data.
